# The BET Inhibitor JQ1 Augments the Antitumor Efficacy of Gemcitabine in Preclinical Models of Pancreatic Cancer

**DOI:** 10.3390/cancers13143470

**Published:** 2021-07-11

**Authors:** Aubrey L. Miller, Patrick L. Garcia, Samuel C. Fehling, Tracy L. Gamblin, Rebecca B. Vance, Leona N. Council, Dongquan Chen, Eddy S. Yang, Robert C. A. M. van Waardenburg, Karina J. Yoon

**Affiliations:** 1 Department of Pharmacology and Toxicology, University of Alabama at Birmingham, Birmingham, AL 35294, USA; aubrey44@uab.edu (A.L.M.); plgarcia@uab.edu (P.L.G.); sfehling@uab.edu (S.C.F.); gamblin@uab.edu (T.L.G.); bvance@uab.edu (R.B.V.); eyang@uab.edu (E.S.Y.); rvanwaar@uab.edu (R.C.A.M.v.W.); 2 Department of Pathology, Division of Anatomic Pathology, University of Alabama at Birmingham, Birmingham, AL 35294, USA; lcouncil@uab.edu; 3 The Birmingham Veterans Administration Medical Center, Birmingham, AL 35233, USA; 4 Department of Medicine, Division of Preventive Medicine, University of Alabama at Birmingham, Birmingham, AL 35205, USA; dchen@uabmc.edu; 5 Department of Radiation Oncology, University of Alabama at Birmingham, Birmingham, AL 35233, USA; 6 O’Neal Comprehensive Cancer Center, University of Alabama at Birmingham, Birmingham, AL 35233, USA

**Keywords:** pancreatic ductal adenocarcinoma, gemcitabine, BET bromodomain inhibitor, JQ1, combination therapy, HMGCS2, APOC1, LXR/RXR, RNA-seq, patient-derived xenograft

## Abstract

**Simple Summary:**

The BET bromodomain inhibitor JQ1 slows tumor growth in preclinical models of pancreatic cancer. However, as a single agent, it does not induce tumor regressions. We hypothesized that JQ1 would sensitize pancreatic tumors to gemcitabine, a drug used for patients with this tumor type. We evaluated the efficacy of JQ1 + gemcitabine in pancreatic cancer cell lines and in two patient-derived xenograft models. The data show that JQ1 + gemcitabine is synergistic in vitro and that this combination has greater efficacy than either drug alone in vivo. RNA-seq analyses to identify mechanisms that may contribute to the observed synergy demonstrated that JQ1 + gemcitabine selectively inhibits expression of proteins necessary for cholesterol biosynthesis and lipid metabolism that support tumor cell proliferation. The data indicate that a BET inhibitor + gemcitabine warrants further investigation for the treatment of pancreatic cancer.

**Abstract:**

Gemcitabine is used to treat pancreatic cancer (PC), but is not curative. We sought to determine whether gemcitabine + a BET bromodomain inhibitor was superior to gemcitabine, and identify proteins that may contribute to the efficacy of this combination. This study was based on observations that cell cycle dysregulation and DNA damage augment the efficacy of gemcitabine. BET inhibitors arrest cells in G1 and allow increases in DNA damage, likely due to inhibition of expression of DNA repair proteins Ku80 and RAD51. BET inhibitors (JQ1 or I-BET762) + gemcitabine were synergistic in vitro, in Panc1, MiaPaCa2 and Su86 PC cell lines. JQ1 + gemcitabine was more effective in vivo than either drug alone in patient-derived xenograft models (*P* < 0.01). Increases in the apoptosis marker cleaved caspase 3 and DNA damage marker γH2AX paralleled antitumor efficacy. Notably, RNA-seq data showed that JQ1 + gemcitabine selectively inhibited HMGCS2 and APOC1 ~6-fold, compared to controls. These proteins contribute to cholesterol biosynthesis and lipid metabolism, and their overexpression supports tumor cell proliferation. IPA data indicated that JQ1 + gemcitabine selectively inhibited the LXR/RXR activation pathway, suggesting the hypothesis that this inhibition may contribute to the observed in vivo efficacy of JQ1 + gemcitabine.

## 1. Introduction

The 5 year survival rate for patients with pancreatic cancer (PC) has remained at less than 10% for decades. Fewer than 20% of patients are eligible for surgery, the only curable treatment. The remaining 80% of patients undergo chemotherapy ± radiation [1]. Combination regimens such as FOLFIRINOX and gemcitabine + nab-paclitaxel are used to treat pancreatic ductal adenocarcinoma (PDAC), the most common type of PC, but are useful only in a subset of patients with good performance status, due to the toxicity of these regimens [2,3,4]. Work presented here focuses on identifying a class of agents that improve the efficacy of gemcitabine-based therapies and that are well tolerated clinically. 

Gemcitabine (2′,2′-difluoro-2′-deoxycytidine; dFdC) is a pyrimidine nucleoside analog with complex pathways of metabolism that contribute to its several mechanisms of action [5,6,7]. A predominant mechanism is the conversion of gemcitabine to dFdCTP, which is incorporated into DNA to inhibit DNA synthesis. Gemcitabine, as a single agent, provides a median survival of ~6 months for PDAC patients [2,4,8]. Combining gemcitabine with agents that dysregulate the cell cycle augments the antitumor efficacy of gemcitabine. For example, paclitaxel causes a G2/M cell cycle arrest and potentiates gemcitabine. This combination is commonly used for the treatment of patients with several types of solid tumors. Agents that increase levels of DNA damage, either by inducing damage or by inhibiting DNA repair, also augment the efficacy of gemcitabine. For example, Chk1 inhibition sensitizes pancreatic cancer cells in vitro to gemcitabine by inhibiting expression of the DNA repair protein RAD51 and sustaining the level of DNA damage induced by gemcitabine [9]. Recent literature focuses on combining gemcitabine with other targeted agents such as inhibitors of CDK4, Wee1, ATR, PLK1, or Aurora B kinase, to effect cell cycle dysregulation [10,11,12,13,14,15].

We previously reported that the BET bromodomain inhibitor JQ1, as a single agent, suppressed PDAC tumor growth in preclinical models and that tumor growth inhibition was concomitant with decreased expression of the G2 cell cycle regulator CDC25B, and increased levels of the DNA damage marker γH2AX [16,17]. The increase in DNA damage most likely results from JQ1-mediated inhibition of expression of the DNA repair proteins Ku80 and RAD51 [17]. We and others show that JQ1 arrests cells in the G1 cell cycle [18,19,20]. Based on these observations, we hypothesized that JQ1-induced cell cycle dysregulation and inhibition of DNA repair would sensitize PDAC cells and tumors to gemcitabine. In this study, we assessed the activity of JQ1 + gemcitabine in vitro and the efficacy of this combination in vivo using three established cell lines and two patient-derived xenograft (PDX) models. We also performed RNA-seq and IPA analyses to identify pathways and gene products that may contribute to the efficacy of the combination. 

## 2. Materials and Methods

### 2.1. Ethics Statement

This study included work performed using vertebrate animals. All procedures were approved by the University of Alabama at Birmingham Institutional Animal Care and Use Committee (IACUC-09186 and -20569).

### 2.2. Cell Lines and Compounds 

Panc1 (CRL-1469), MiaPaCa2 (CRL-1420), and Su86 (CRL-1837; SU.86.86) pancreatic cancer cell lines were purchased from the American Type Culture Collection (Manassas, VA, USA). All cells were cultured under the recommended conditions. All cell lines used were tested for mycoplasma using MycoAlertTM PLUS Mycoplasma Detection Kit (Lonza, Walkersville, MD, USA), and the results were negative. Gemcitabine hydrochloride (G-4177, LC Laboratories, Woburn, MA, USA) was dissolved in sterile phosphate-buffered saline or 0.9% saline solution for in vitro and in vivo experiments, respectively. JQ1 (HY-13030, MedChem Express, Monmouth Junction, NJ, USA) and I-BET762 (MedChem Express) were dissolved in DMSO. DMSO concentration was <0.1% in vitro. 

### 2.3. In Vitro Cell Viability Assay

Cell viability assays were performed as previously described [17]. Briefly, PDAC cells were seeded in 96-well plates and allowed to adhere overnight. Serial dilutions of BET inhibitor and/or gemcitabine were prepared and added to wells for 72 h. AlamarBlue (Fisher Scientific, Waltham, MA, USA) reagent was added to the culture medium according to the manufacturer’s recommendation. Fluorescence was read on a Victor X5 microplate reader at 590 nm. 

### 2.4. Clonogenic Assay

Clonogenic assays were performed as previously described [17]. Briefly, cells were seeded in 24-well plates, allowed to attach overnight and exposed to DMSO, JQ1 (250 nM) gemcitabine (25 nM), or JQ1 + gemcitabine for 72 h. The medium was removed, cells were washed with PBS, and drug-free medium was added. The cells were incubated for an additional 11 days, after which they were fixed and stained with 0.25% crystal violet. Colonies containing >50 cells were counted. 

### 2.5. Immunoblot Analysis

Whole-cell lysates were prepared in NP-40 lysis buffer (Boston BioProducts, Ashland, MA, USA), with protease inhibitor cocktail (Fisher Scientific). Immunoblot analysis of lysates was carried out using standard methods [17]. Primary antibodies used were: γH2AX (#9718, Cell Signaling, Danvers, MA, USA), cleaved PARP (#5625, Cell Signaling), and α-tubulin (#2125, Cell Signaling). Immunoblots were quantitated using ImageJ (1.15 s, National Institutes of Health, Bethesda, MD, USA).

### 2.6. In Vivo Drug Efficacy

Four-week-old female SCID CB 17^-/-^ mice purchased from Taconic Farms (Newton, MA, USA) or Charles River (Wilmington, MA, USA) were housed in the AAALAC accredited vivarium at UAB Research Support Building under barrier conditions, with a 12 h light/dark cycle and access to food and water ad libitum. Development and characterization of PDAC patient-derived xenografts UAB-PA4 and UAB-PA16 (hereafter, PA4 and PA16) used in this study have been reported [21]. When tumors reached ~200 mm^3^, mice bearing bilateral tumors were randomized to 5 mice/group with the exception of the vehicle control (VC) and JQ1 groups for PA16, which had 4 mice/group (8–10 tumors/group; PA4 had 10 tumors/group and PA16 had 8 [VC, JQ1] or 9 [gem, JQ1+gem] tumors/group). We administered VC (saline followed by 10% DMSO in 10% β-cyclodextrin), 100 mg/kg gemcitabine weekly, 50 mg/kg JQ1 daily, or a combination (gemcitabine 100 mg/kg weekly followed by 50 mg/kg JQ1 daily) by intraperitoneal injection for 21 days. Tumor size was measured with Vernier calipers three times a week, and tumor volumes calculated using the equation v = (π/6)*d^3^. Twenty-four hours following final treatment, mice were euthanized by CO_2_ inhalation followed by cervical dislocation, tumor tissue harvested, and either formalin fixed and paraffin embedded or snap frozen in liquid nitrogen for further analysis. Tumor volumes are represented as the mean ± SEM and were analyzed and compared using two-way analysis of variance (ANOVA) with GraphPad Prism (v.7) (San Diego, CA, USA). 

### 2.7. Hematoxylin and Eosin Staining

Histological analysis was performed as previously described [16,17,21]. An Olympus BH-2 microscope with DP71 camera and DPS-BSW v3.1 software (Center Valley, PA, USA) was used to take photomicrographs. Archived images were read in the Department of Pathology by LNC.

### 2.8. Immunohistochemistry

Immunohistochemical staining and analyses were performed as previously described [16,17,21]. Briefly, Ki67, γH2AX, and cleaved caspase 3 indices were calculated by counting the number of positive tumor cells in ten random high-magnification fields and dividing by the total number of tumor cells in those fields. Data are presented as the mean ± SEM of two-independent experiments. Expression indices were calculated by assigning a staining intensity (0, 1, 2, 3) for all stained tumor cells [22]. The percentage of positive cells was multiplied by the staining intensity to give values ranging from 0 to 300. Photomicrographs were taken on an Olympus BH-2 microscope with DP71 camera and DPS-BSW v3.1 software (Center Valley, PA, USA) and on a Zeiss Axio Observer Z.1 microscope with the Zen 2 Blue imaging software. Primary antibodies: Ki67 (ab92742, abcam, Waltham, MA, USA), γH2AX (#9718, Cell Signaling, Danvers, MA, USA), cleaved caspase 3 (#9661, Cell Signaling), APOC1 (ab198288, abcam), and HMGCS2 (PA5-55620, Fisher Scientific, Waltham, MA, USA). 

### 2.9. RNA Isolation and RNA-Sequencing

Total RNA was isolated from snap-frozen tumor tissue specimens using TRIzol-chloroform extraction. RNA was processed for sequencing using the RNeasy Mini Kit (Qiagen, Germantown, MA, USA). Purified RNA concentration and quality was determined by ND-1000 spectrophotometer using NanoDrop 3.0.1 software (Coleman Technologies Inc., Wilmington, DE, USA). RNA samples (200 ng) were analyzed at the UAB Heflin Center Genomics Core (Birmingham, AL, USA). The RNA-seq libraries were prepared on the NextSeq500 Illumina Sequencing Platform (Illumina, San Diego, CA, USA) with paired end 75 bp at 40M reads. 

### 2.10. Bioinformatics 

Raw data were obtained from the Sequencing Core Lab in fastq format and processed by first removing adapter sequences using Trimmomatic tool [23], and then aligning with the HISAT2 algorithm [24]. PCR replicates were removed using SAMtools [25], and the resulting BAM or SAM files used to quantitate expression level, using Partek Genomics Suite (PGS, St. Louis, MO, USA). Normalization of reads per kilobase of transcripts per million mapped reads (RPKM) was performed before statistical analysis to quantitate fold-changes of specific genes and *P* values [26]. Candidate genes with fold changes ≥2 between specific experimental cohorts underwent additional pathway analysis using Ingenuity Pathway Analysis (IPA, Redwood City, CA USA). The RNA sequencing data presented in this study are openly available in gene expression omnibus (GEO) database at [ncbi.nlm.nih.gov/geo] accessed on 14 June 2021, reference number (GSE174023) and in Supplementary Materials (Excel S1). 

### 2.11. Real-Time PCR, qRT-PCR

qRT-PCR was performed as previously described [16,17]. Primers used are listed in Table S1.

### 2.12. Statistics

All statistical analyses for in vitro and in vivo assays except RNA-seq analysis were performed using GraphPad Prism 7 (San Diego, CA, USA). In vitro data were analyzed by one-way ANOVA followed by a Tukey post-hoc test or Student’s *t* test. In vivo data were analyzed by two-way ANOVA followed by a Tukey post-hoc test. *P* < 0.05 was considered significant. All in vitro assays were performed a minimum of three times. 

## 3. Results

### 3.1. The Combination of a BET Inhibitor + Gemcitabine Exerts Synergistic Cytotoxicity in PDAC Cell Lines

**Figure 1:**
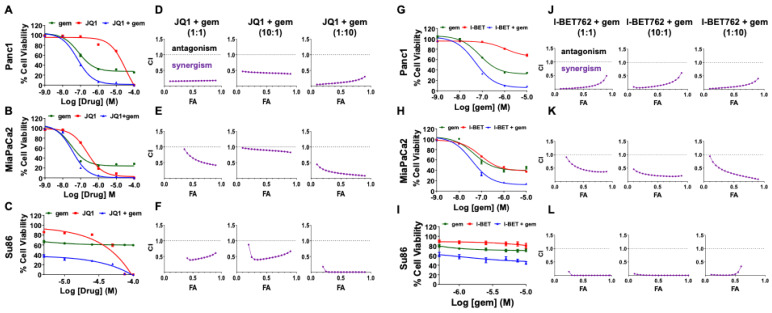
A BET inhibitor (JQ1 or I-BET762) + gemcitabine induces synergistic cytotoxicity in PDAC cell lines. Panc1, MiaPaCa2, and Su86 cells were exposed to various concentrations of JQ1 (0.001–100 µM) and gemcitabine (0.001–100 µM) in a 1:1 ratio (**A**–**C**), or I-BET762 (0.01–100 µM) and gemcitabine (0.001–10 µM) in a 10:1 ratio (**G**–**I**) for 72 h, and cell viability quantitated using alamarBlue assays. Data are normalized to controls and are presented as the mean ± SEM. (**D**–**F**,**J**–**L**) Combination indices of <1.0 demonstrate that both combinations are synergistic at all three ratios (1:1, 10:1, and 1:10) and in all three cell lines. Combination indices (CI) are plotted against fraction of cells affected (FA) using compuSyn software.

As single agents, JQ1 and gemcitabine inhibit PDAC tumor growth in in vivo PDX models [16,17,27]. We proposed that JQ1-induced cell cycle arrest, coincident with increases in levels of DNA damage, would sensitize PDAC tumor cells to gemcitabine. To address this hypothesis, we performed alamarBlue cell viability assays to evaluate the effect of the combination on Panc1, MiaPaCa2, and Su86 pancreatic cancer cell lines in vitro. Cells were exposed to a range of concentrations of JQ1 and gemcitabine in ratios of 1:1, 10:1, or 1:10 for 72 h. Cells exposed to JQ1 only, gemcitabine only, or vehicle only served as controls. The data show that JQ1 or gemcitabine reduced cell viability in all cell lines, and the two drugs together were more effective than either drug alone (Figure 1A–C). Combination indices (CI), calculated using compuSyn, indicated synergy for JQ1 + gemcitabine for all three cell lines, irrespective of ratio (Figure 1D–F). To then address whether the observed synergy was specific for JQ1, we evaluated a second BET inhibitor, I-BET762, in combination with gemcitabine. I-BET762 was not as potent as JQ1 as a single agent, but synergy was also observed with I-BET762 + gemcitabine (Figure 1G–I). CI values indicated synergy for all ratios evaluated in all three cell lines (Figure 1J–L). 

### 3.2. JQ1 + Gemcitabine Decreases Colony Formation In Vitro, Concomitant with Increases in Levels of the DNA Damage Marker γH2AX and the Apoptosis Marker Cleaved PARP 

**Figure 2:**
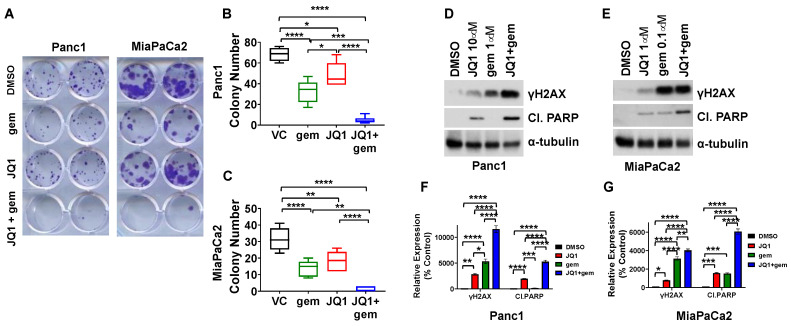
JQ1 + gemcitabine inhibits colony formation, increases levels of DNA damage marker γH2AX and increases apoptosis marker cleaved PARP in PDAC cells. (**A**) Panc1 and MiaPaCa2 cells were exposed to DMSO (vehicle control, VC), gemcitabine (25 nM), JQ1 (250 nM), or the combination for 72 h, and then incubated for an additional 11 days in drug-free medium. (**B**,**C**) Colonies of >50 cells were quantitated, using standard methods. Data are analyzed using one-way ANOVA (* *P* < 0.05, ** *P* < 0.01, *** *P* < 0.001, **** *P* < 0.001), and are presented as box and whisker plots. (**D**,**E**) Immunoblots were performed to detect γH2AX and cleaved PARP (Cl. PARP) following 48 h exposure to DMSO, JQ1, gemcitabine, or the combination in Panc1 (**D**) and MiaPaCa2 (**E**) cells. (**F**,**G**) Immunoblot data were quantitated as percent control using ImageJ and are reported as bar graphs of mean ± SEM. Uncropped immunoblot images with densitometry intensity/readings are shown in Supplementary Materials (Figures S4 and S5). Analysis was performed by one-way ANOVA (* *P* < 0.05, ** *P* < 0.01, *** *P* < 0.001, **** *P* < 0.0001).

We also determined the effect of JQ1 + gemcitabine on the clonogenic potential of Panc1 and MiaPaCa2 cells (Figure 2A). Cells were exposed to JQ1 + gemcitabine (10:1) for 72 h and then incubated in drug-free medium for an additional 11 days. JQ1 + gemcitabine decreased colony formation from >30 to 0–3 (*P* < 0.0001) for MiaPaCa2 and from >70 to 2–11 for Panc1 (*P* < 0.0001). The combination was more effective than either drug alone (*P* < 0.05) (Figure 2A–C). Of note, Su86 cells did not form colonies in this assay.

The decrease in clonogenic potential in Panc1 and MiaPaCa2 cells was concomitant with an increase in the level of the DNA damage marker γH2AX in Panc1 and MiaPaCa2 cells and an increase in the apoptosis marker cleaved PARP, compared to DMSO (*P* < 0.0001) or either drug as a single agent (*P* < 0.01, *P* < 0.0001) (Figure 2D–G). 

### 3.3. JQ1 + Gemcitabine Suppresses PDAC Tumor Growth In Vivo

**Figure 3:**
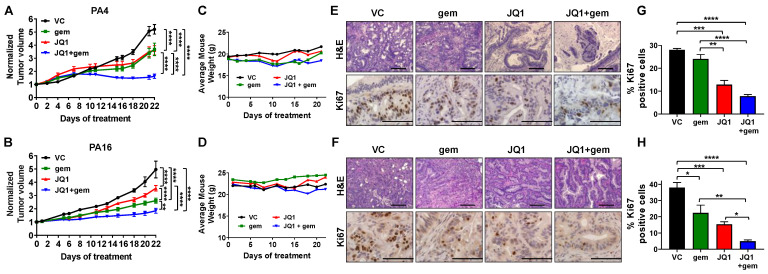
The combination of JQ1 + gemcitabine inhibits the growth of PA4 and PA16 patient-derived xenograft models of PDAC. (**A**,**B**) Tumor-bearing mice were treated with vehicle (VC), JQ1 (50 mg/kg daily), gemcitabine (gem, 100 mg/kg weekly) or JQ1 + gemcitabine (JQ1+gem) for 21 days. Tumor volume was measured three times a week and data for PA4 (**A**) and PA16 (**B**) tumors are presented as normalized volume ± SEM. Two-way ANOVA analysis indicated that the combination was more effective than either drug as a single agent (** P < 0.01, **** *P* < 0.0001). (**C**,**D**) The average mouse weight for mice bearing PA4 (**C**) and PA16 (**D**) did not change during this study. Tumor tissue was harvested 24 h following the final treatment. (**E**,**F**) FFPE sections from PA4 (**E**) and PA16 (**F**) were stained with H&E and immunostained to detect the proliferation marker Ki67. Scale bar 10x = 20 µm and 40x = 10 µm. (**G**,**H**) Ki67 data were quantitated as detailed in Methods, and are presented as the mean ± SEM. One-way ANOVA analysis was performed (* *P* < 0.05, ** *P* < 0.01, *** *P* < 0.001, **** *P* < 0.0001).

We next evaluated the efficacy of JQ1 + gemcitabine in vivo, in two PDX models that were independently derived from primary human tumors: PA4 and PA16. Cohorts of tumor-bearing mice were treated with vehicle, JQ1 (50 mg/kg daily x 21), gemcitabine (100 mg/kg weekly x 3) or JQ1 + gemcitabine. The combination was more effective than vehicle (*P* < 0.0001), JQ1 alone (*P* < 0.0001) or gemcitabine alone (*P* < 0.01) in both models (Figure 3A,B). Actual tumor volumes are shown in Figure S1. Notably, JQ1 + gemcitabine induced stable disease in the PA4 model. All mice maintained body weight within 10% of initial weight (Figure 3C,D). Although we observed all mice maintained body weight throughout the experiment, the safeness of these treatments in vivo needs to be further evaluated using additional analyses including hematopoietic, liver, and kidney function.

Twenty-four hours following completion of therapy, mice were euthanized and tumors were harvested. Tumor histology was assessed by staining FFPE tissue sections with H&E (Figure 3E,F); proliferative index was assessed by immunostaining for Ki67. Tumor histology indicated that JQ1 and JQ1 + gemcitabine induced tumor cell differentiation in both models (Figure S2). PA4 tumors and PA16 tumors exposed to JQ1 or JQ1 + gemcitabine had a more well-differentiated phenotype with intact nuclear polarity, less nuclear stratification, and a lower N:C ratio compared to vehicle controls or tumors treated with gemcitabine alone. FFPE tissue sections immunostained to detect the tumor cell proliferation marker Ki67 showed that compared to vehicle controls and gemcitabine alone, JQ1 + gemcitabine decreased the Ki67 index ~3- to 8-fold in both models (*P* < 0.05) (Figure 3E–H). 

### 3.4. Tumors Exposed to JQ1 + Gemcitabine In Vivo Have Higher Levels of the DNA Damage Marker γH2AX and the Apoptosis Marker Cleaved Caspase 3 Than Either Drug as a Single Agent

**Figure 4:**
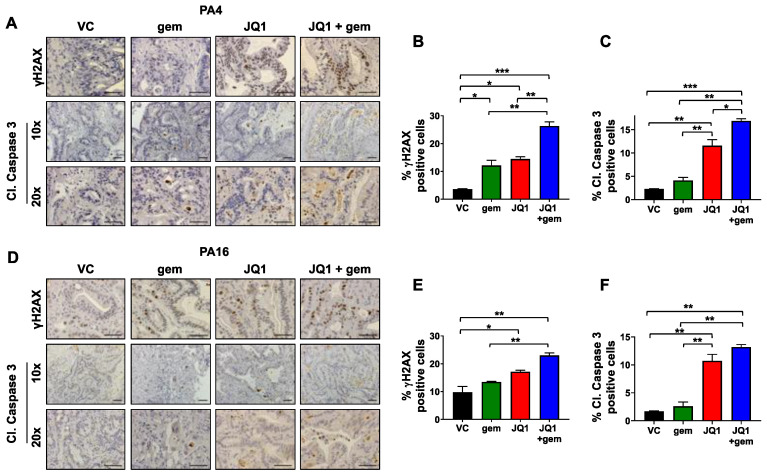
JQ1 + gemcitabine increases levels of DNA damage marker γH2AX and apoptosis marker cleaved caspase 3 in PDAC tumors in vivo. (**A**,**D**) FFPE sections from PA4 (**A**) and PA16 (**D**) tumor models were immunostained to detect the DNA damage marker γH2AX and the apoptosis marker cleaved caspase 3. Scale bar = 100 µm. (**B**,**C**,**E**,**F**) Quantitation of the γH2AX and cleaved caspase 3 (Cl. Caspase 3) results for PA4 (**B**,**C**) and PA16 (**E**,**F**) tumors are shown as bar graphs. Data are presented as the mean ± SEM and analyzed using one-way ANOVA (* *P* < 0.05, ** *P* < 0.01, *** *P* < 0.001).

The literature documents that gemcitabine induces DNA damage by incorporation into DNA as a modified nucleotide [7]. We have shown that JQ1 increases levels of DNA damage, as assessed by levels of γH2AX, likely by inhibiting expression of DNA repair proteins RAD51 and Ku80 [17]. We then asked whether DNA damage induced by or sustained by the combination was greater than either drug as a single agent. IHC data using tumor tissue harvested from mice treated with JQ1, gemcitabine, or the combination showed that all three regimens increased levels of γH2AX in vivo (*P* < 0.05) (Figure 4A,D). In PA4, more DNA damage was evident after exposure to the combination than to either drug alone (*P* < 0.01) (Figure 4A,B). In PA16, DNA damage by the two agents was greater than gemcitabine alone (*P* < 0.01) (Figure 4D,E). Immunostaining of FFPE sections showed that JQ1 and JQ1 + gemcitabine mediated 5- to 7-fold and 6- to 8-fold increases in PA4 and PA16 tumors, respectively, in the apoptosis marker cleaved caspase 3 (Figure 4A,C,D,F). Apoptotic regions were observed predominently in sections that included the outer edge of tumors. 

### 3.5. JQ1 + Gemcitabine Inhibits the Expression of Gene Products of the Lipid Metabolism and the LXR/RXR Activation Pathway 

Because JQ1 inhibits an early stage of transcription of genes whose expression is BET dependent, we wanted to identify gene products whose expression was inhibited by JQ1 + gemcitabine that might contribute to the efficacy of this combination. We extracted RNA from tumors of vehicle treated mice (VC) and from tumors exposed to JQ1+ gemcitabine in vivo, and performed RNA-seq analysis (UAB Heflin Center, Birmingham, AL, USA). 

Compared to tumors exposed to vehicle only, JQ1 + gemcitabine decreased expression of 68 genes in the PA4 model and 1,109 genes in the PA16 model by >2-fold. Only eight RNA species were downregulated in both models: *HMGCS2*, *APOC1*, *GBP4*, *ARL4C*, *CGBS*, *NEGR1*, *PNCK* and *TAC3* (Table S2). The finding that JQ1 + gemcitabine decreased expression of RNA-encoding HMGCS2 and APOC1 by ~6-fold and 3- to 8-fold, respectively, has not been reported previously. HMGCS2 (3-Hydroxy-3-Methylglutaryl-CoA Synthase 2) and APOC1 (Apolipoprotein C1) have multiple cellular functions; both contribute to lipid metabolism and homeostasis. Specific effects of these proteins relevant to tumor progression are discussed below. 

We then corroborated RNA-seq data for HMGCS2 and APOC1 by qRT-PCR and used IHC staining to determine whether RNA levels reflected protein levels. We also performed Ingenuity Pathway Analysis (IPA) to evaluate whether the observed decrease in HMGCS2 and APOC1 reflected a change in the activity or function of a specific pathway, with particular interest in pathways that contribute to lipid metabolism. 

**Figure 5:**
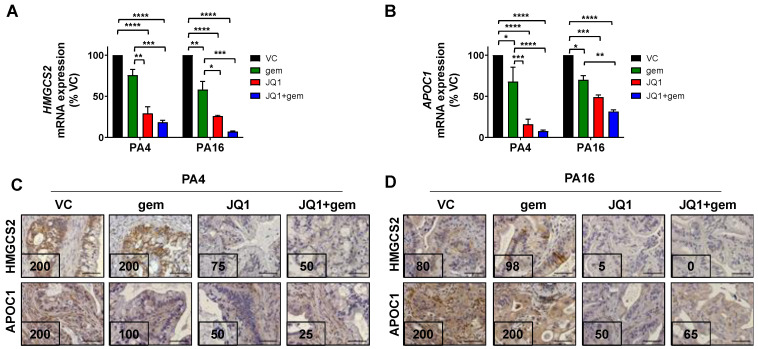
JQ1 + gemcitabine inhibits the expression of HMGCS2 and APOC1 in PA4 and PA16 tumors. (**A**,**B**) qRT-PCR analysis for HMGCS2 (**A**), and APOC1 (**B**) mRNA expression in PA4 and PA16 tumors harvested from mice treated with vehicle (VC), JQ1, gem, or JQ1+gem. Data are presented as the mean ± SEM. One-way ANOVA followed by a Tukey post- hoc test were performed (* P < 0.05, ** *P* < 0.01, *** *P* < 0.001, **** *P* < 0.0001). FFPE tumor sections of PA4 (**C**) and PA16 (**D**) were immunostained to detect HMGCS2 and APOC1 protein expression. Expression indices are shown in the lower left-hand corner of each photomicrograph and were calculated as described in Materials and Methods. Scale bar = 100 µm.

qRT-PCR analysis for HMGCS2 and APOC1 confirmed RNA-seq data: JQ1 + gemcitabine decreased mRNA levels of HMGCS2 and APOC1 by ~82% and ~92% in PA4 and by ~93% and ~69% in PA16 (*P* < 0.0001), respectively (Figure 5A,B). Important to the relevance of observed changes in RNA levels, IHC staining demonstrated that JQ1 + gemcitabine decreased levels of HMGCS2 protein by 4- and 8-fold and of APOC1 by 8- and 3-fold in PA4 and PA16 tumors, respectively (Figure 5C,D). 

**Figure 6:**
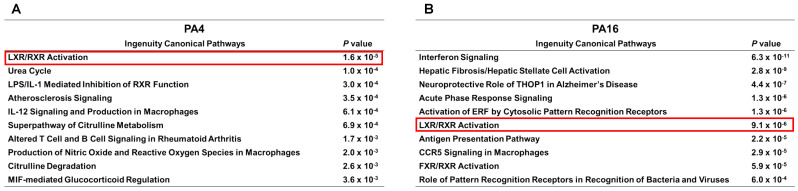
The LXR/RXR activation pathway (red frame) is among the top pathways downregulated by JQ1 + gemcitabine in vivo in PDAC tumor models. (**A**,**B**) RNA extracted from snap-frozen tissue from tumors of mice treated with vehicle (VC) and JQ1 + gemcitabine were analyzed by RNA-seq and Ingenuity Canonical Pathway Analysis to identify the ten most affected pathways in PA4 (**A**) and PA16 (**B**) tumors. *P* values reflect difference in tumors exposed in vivo to JQ1 + gemcitabine compared to vehicle only.

We reasoned that if the observed changes in these two proteins had functional impact on lipid metabolism, IPA of RNA-seq data would reflect a change in a lipid-associated pathway(s) (Figure 6). Indeed, IPA identified only one pathway that was affected in both PA4 and PA16 tumors exposed in vivo to JQ1 + gemcitabine: the liver X receptor (LXR)/retinoid X receptor (RXR) activation pathway which regulates lipid metabolism (*P* values 9.11 × 10^−6^ and 1.6 × 10^−5^ for PA16 and PA4, respectively) (Figure 6A,B). Table S3 shows gene products of the LXR/RXR activation pathway downregulated following exposure to JQ1 + gemcitabine, compared to controls. APOC1 was one of the most downregulated by this combination: by 8-fold and 3-fold in PA4 and PA16 tumors, respectively (Table S3). In contrast, no gene products or pathways were upregulated in both models (Figure S3). 

The data indicate that the pathway most affected by exposure to JQ1 + gemcitabine was the LXR/RXR lipid metabolic pathway, and that JQ1 and JQ1 + gemcitabine decreased expression of HMGCS2 and APOC1, which contribute to cholesterol biosynthesis and lipid homeostasis. 

## 4. Discussion

This study addressed the hypothesis that the cell cycle abrogation and increased levels of DNA damage marker induced by BET inhibitors would sensitize PDAC cells and tumors to gemcitabine. The BET inhibitor JQ1 + gemcitabine was synergistic in vitro and more effective than either drug as a single agent in vivo in two models (*P* < 0.01 and *P* < 0.001). The principal novel finding of the work is the identification of specific proteins and pathways that have functional relevance in PDAC tumor growth and progression and to sensitivity to gemcitabine. More specifically, the data suggest that alterations in lipid metabolism may contribute to or even regulate PDAC tumor growth, and that interrupting this pathway may be an effective approach to treatment for patients with PDAC tumors. Additional novel findings include the following. (1) We are the first to show synergistic cytotoxicity by a BET inhibitor + gemcitabine. (2) We are the first to use multiple patient-derived xenograft models of PDAC to evaluate efficacy. (3) We are the first to use RNA-seq transcriptomic and IPA analyses for models of human origin exposed in vivo to JQ1 + gemcitabine, to identify gene products and pathways up- or downregulated by this combination in PDAC PDX tumors. Because BET inhibitors decrease expression of many gene products, transcriptomic analyses were performed to identify pathways and gene products that might contribute to the in vivo efficacy of this combination. The data demonstrate inhibition of HMGCS2 and APOC1 expression and decreases in gene products of the LXR/RXR activation pathway. We propose that proteins that contribute to cholesterol biosynthesis, lipid homeostasis, and the LXR/RXR pathway represent potential therapeutic targets for the treatment of PDAC.

PA4 and PA16 PDX PDAC models were derived from primary human pancreatic tumors that express G12D KRAS, a mutated protein expressed >90% of primary PDAC tumors [21]. These in vivo models responded similarly to JQ1, gemcitabine and JQ1 + gemcitabine. We envisioned that expression profiling would identify molecules relevant to the efficacy of a given regimen, and that relevant changes in expression following drug exposure would be limited to molecules affected in both models. The unexpected finding was that only eight gene products were observed to be expressed at >2-fold higher levels in tumors exposed in vivo to JQ1 + gemcitabine compared to controls in both models. This observation facilitated mechanistic investigations since it significantly restricted the number of ‘genes of interest’, and pathway analysis demonstrated a likely association of lipid metabolism to PDAC tumor progression and to the efficacy of JQ1 + gemcitabine.

Our findings for the efficacy of a BET inhibitor + gemcitabine with patient-derived models are consistent with data in the literature that were generated using cell line-derived xenograft models and GEM models [28,29]. Mazur et al. reported that JQ1 + gemcitabine slowed tumor growth (*P* < 0.05) more than either drug as a single agent in GEM mutant Kras, p53 mice, and the combination increased duration of overall survival compared to control or gemcitabine only groups (*P* < 0.001) [28]. Xie et al., demonstrated that gemcitabine + I-BET762 was superior to either drug alone in models of cell line-derived xenografts [29]. 

Recent studies in the literature demonstrate that JQ1 in combination with the PARP inhibitor olaparib, the immunotherapeutic agent α-PD-L1, the HDAC inhibitor SAHA, or arsenic trioxide are more effective than these drugs as single agents in in vivo models of pancreatic cancer [17,28,30,31]. Our laboratory (2019) showed that JQ1 decreased expression of DNA repair proteins RAD51 and Ku80, and sensitized PDAC patient-derived xenograft tumors to the PARP inhibitor olaparib. This combination was also more effective than either drug alone [17]. Data from that study are consistent with the hypothesis that the synergy of the combination reflects observations that DNA repair deficient tumor cells are relatively sensitive to olaparib. Taken together, the literature documents an emerging role for BET inhibitors as ‘sensitizing agents’ to augment the efficacy of agents of other mechanistic classes.

Our findings that JQ1 + gemcitabine decreased two proteins (HMGCS2 and APOC1) that impact cholesterol biosynthesis and lipid metabolism and inhibited the LXR/RXR activation pathway were unexpected and have not been reported previously. However, this finding aligns with recent publications documenting the involvement of lipid metabolism on sensitivity to gemcitabine as a single agent [32,33,34]. For example, El Kaoutari et al. (2021) generated metabolomic profiles of 77 pancreatic cancer patient-derived tumor models. They then correlated profile data from 35 patient-derived primary tumor cell lines with in vitro sensitivity to five drugs including gemcitabine, and expressed the data as a resistance score (GRM). A notable finding from that study was that resistance scores correlated with duration of survival of patients from whom tumor specimens were obtained. In that study, resistance to gemcitabine was associated with increases in glycerophospholipids and decreases in lysophospholipids [32]. Together, these findings suggest strongly that lipid metabolism impacts PDAC tumor progression and the efficacy of JQ1 + gemcitabine. Our data suggest that HMGCS2 and APOC1, regulators of lipid metabolism, represent potential drug targets. 

HMGCS2 and APOC1 have multiple functions. HMGCS2 is a mitochondrial enzyme that is rate-limiting in ketogenesis and essential in converting acetoacetyl-CoA to HMG-CoA, a cholesterol precursor [35,36]. With respect to tumor cell proliferation and tumor growth, in vitro studies have shown that HMGCS2 downregulation inhibits prostate cancer cell proliferation in 2D cultures and of 3D spheroid growth [37]. Additionally, in vitro, co-culture of prostate cancer cells with cancer-associated fibroblasts upregulates expression of multiple genes of the cholesterol biosynthesis pathway, with HMGCS2 being the most upregulated [37]. Chen et al. demonstrated that HMGCS2 facilitated colorectal and oral cancer cell invasion and migration by interaction with PPARα and activation of Src signaling in vitro [38]. Further, colorectal tumor cells transfected with shHMGCS2 formed fewer metastatic lesions and host mice survived longer (*P* = 0.0017), compared to control transfectants. These investigators suggested that HMGCS2 may be a therapeutic target in colorectal or oral cancer. In contrast, Wang et al. showed that HMGCS2 knockdown supported hepatocellular carcinoma cell proliferation and migration [39]. Comparison of these data suggest that the function of HMGCS2 may depend on tumor cell type. The role of HMGCS2 in PDAC has not been studied. 

A second protein we determined to be decreased by JQ1 + gemcitabine is APOC1. APOC1 is expressed predominantly in liver. One of its well characterized functions is to inhibit lipoprotein binding to very low density lipoprotein (VLDL) receptor and low density lipoprotein (LDL) receptor, with a consequent increase in serum cholesterol and triglycerides [40,41]. APOC1 has been observed to be overexpressed in colorectal, lung, pancreatic cancer and renal cell carcinoma. APOC1 expressed at relatively high levels is a negative prognostic indicator and correlates with poor overall survival for patients with clear cell renal cell carcinoma [42,43,44]. Recent literature documents the role of APOC1 in several in vitro and in vivo tumor models. For example, APOC1 facilitates clear cell renal cell carcinoma metastasis by STAT3 pathway activation [43]. APOC1 is also reported to promote colorectal cancer proliferation, migration and invasion by activating the MAPK pathway [44]. 

Thirdly, LXR and RXR are nuclear receptors that heterodimerize to increase de novo lipogenesis by complex mechanisms [45], and our IPA analyses demonstrate that JQ1 + gemcitabine decreases expression of multiple gene products of the LXR/RXR activation pathway in vivo. A few published studies address the function of LXR in pancreatic cancer cells, but the data from these studies are inconsistent. Candelaria et al. reported that activation of the LXR pathway inhibited PDAC cell proliferation in vitro [46]. In contrast, and in agreement with our findings, Karaboga et al. found that inhibiton of the LXR activity inhibited PDAC cell proliferation in vitro [47]. Studies using in vitro models of prostate, colorectal, clear cell renal carcinoma, and lung cancer cells lines have also found that inhibition of components of the LXR/RXR pathway decreases cell viability [44,45,46]. Data generated with in vitro and in vivo models of breast cancer also do not lead to a definitive conclusion regarding a direct vs. an inverse relationship between activation or inhibition of the LXR/RXR activation and breast cancer cell viability [48,49,50]. For example, Carpenter et al., reported that inhibition of LXR inhibited triple negative breast tumor growth, by increasing CD8+ T cell activity [48]. In contrast, activation of LXR by LXR agonist inhibited breast cancer cell proliferation in vitro [51]. Interestingly, Hutchinson et al. reported that activation of LXR by LXR antagonist contributes to resistance of triple negative breast cancer to epirubicin in vivo [52]. Possibly, the efficacy of specific agents depends on in vitro vs. in vivo methods, tumor type or genotype, or the specific regimen or drug combination evaluated. Ongoing work from several laboratories with agents that activate (GW3965, T09031317) or inhibit (GSK2033, SR9243) LXR activity are being evaluated in preclinical models of melanoma, colorectal, and small-cell lung cancer [53,54,55]. Our data indicate that decreased expression of components of LXR/RXR function is co-incident with inhibition of tumor progression in preclinical PDAC models. 

## 5. Conclusions

In vitro, JQ1 + gemcitabine was synergistic in PDAC cell lines. In vivo, this combination had greater efficacy than either drug alone in two independently derived PDAC PDX models (*P* < 0.01). RNA-seq, qRT-PCR and IPA analyses suggest that decreased expression of HMGCS2 and APOC1 and decreases in components of the LXR/RXR activation pathway may contribute to the efficacy of JQ1 + gemcitabine. Current work focuses on identifying mechanisms by which a BET inhibitor + gemcitabine inhibits PDAC tumor progression.

## Data Availability

The RNA sequencing data presented in this study are openly available in gene expression omnibus (GEO) database at [ncbi.nlm.nih.gov/geo] accessed on 14 June 2021, reference number (GSE174023).

## Supplementary Materials

The following are available online at https://www.mdpi.com/article/10.3390/cancers13143470/s1, Figure S1: JQ1 + gemcitabine inhibits tumor growth of PA4 and PA16 PDX models of PDAC in vivo; Figure S2: JQ1 and JQ1 + gemcitabine induce tumor cell differentiation in vivo; Figure S3: Pathways and gene products upregulated by JQ1 + gemcitabine in vivo in PA4 or PA16 PDX models of PDAC; Figure S4: The whole uncropped immunoblot images with densitometry intensity/reading analysis of Figure 2D; Figure S5: The whole uncropped immunoblot images with densitometry intensity/reading analysis of Figure 2E; Table S1: Primers used to perform qRT-PCR; Table S2: Eight gene products downregulated in vivo by JQ1 + gemcitabine compared to vehicle control in PA4 and PA16 PDAC tumors; Table S3: Gene products in the LXR/RXR activation pathway that are altered by JQ1 + gemcitabine compared to vehicle control in PA4 and PA16 tumors; Excel S1: RNA-seq data for up- or downregulated genes by JQ1 + gemcitabine compared to control.
