# Supplementary material for: The BET Inhibitor JQ1 Augments the Antitumor Efficacy of Gemcitabine in Preclinical Models of Pancreatic Cancer
Source: Cancers (Basel). 2021 Jul 11;13(14):3470. doi: 10.3390/cancers13143470 (PMC8303731; doi:10.3390/cancers13143470)
Supplement: Supplementary file 1 [file cancers-13-03470-s001.zip › cancers-1278814-supplementary/cancers-1278814 Supplementary/cancers-1278814supplementary.v3.pdf]

cancers

MDPI

The BET inhibitor JQ1 augments the antitumor efficacy of gemcitabine in preclinical models of pancreatic cancer

Aubrey L. Miller, Patrick L. Garcia, Samuel C. Fehling , Tracy L. Gamblin , Rebecca B. Vance , Leona N. Council, Dongquan Chen, Eddy S. Yang, Robert C. A. M. van Waardenburg, and Karina J. Yoon

Supplementary Materials

Figure S1. JQ1 + gemcitabine inhibits tumor growth of PA4 and PA16 PDX models of PDAC *in vivo*.

Figure S2. JQ1 and JQ1 + gemcitabine induce tumor cell differentiation *in vivo*.

Figure S3. Pathways and gene products upregulated by JQ1 + gemcitabine *in vivo* in PA4 or PA16 PDX models of PDAC.

Figure S4. The whole uncropped immunoblot images with densitometry intensity/reading analysis of Figure 2D.

Figure S5. The whole uncropped immunoblot images with densitometry intensity/reading analysis of Figure 2E.

Table S1. Primers used to perform qRT-PCR.

Table S2. Eight gene products downregulated *in vivo* by JQ1 + gemcitabine compared to vehicle control in PA4 and PA16 PDAC tumors.

Table S3. Gene products in the LXR/RXR activation pathway that are altered by JQ1 + gemcitabine compared to vehicle control in PA4 and PA16 tumors.

Excel S1. RNA-seq data for up-or down-regulated genes by JQ1 + gemcitabine compared to control.

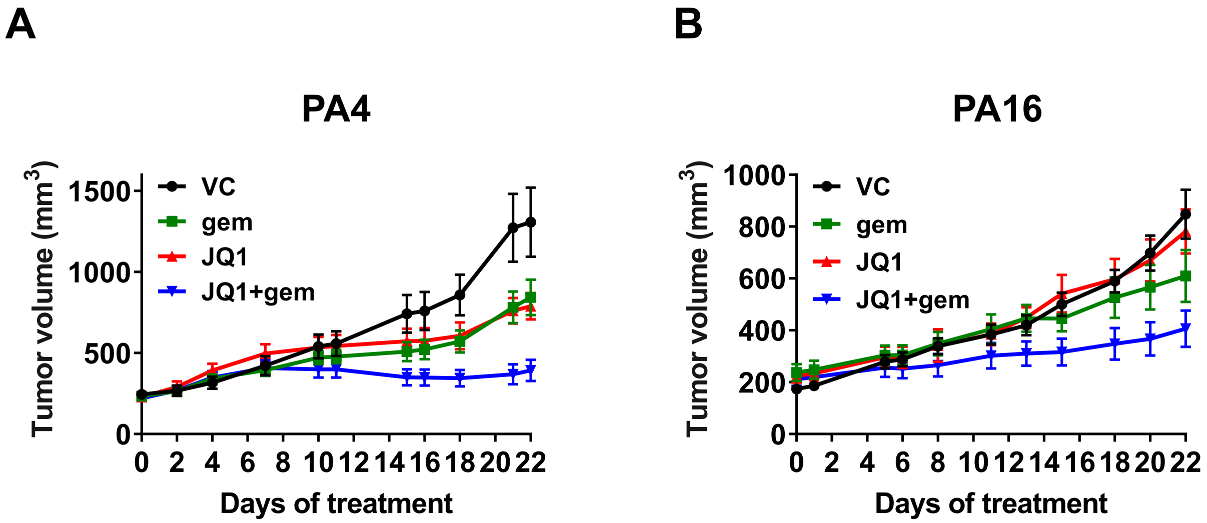

Figure S1. JQ1 + gemcitabine inhibits tumor growth of PA4 (A) and PA16 (B) patient-derived xenograft models of PDAC *in vivo*. Tumor bearing mice were treated with Vehicle Control (VC), JQ1 (50mg/kg daily), gemcitabine (gem; 100mg/kg weekly), or JQ1 + gemcitabine (JQ1+gem) for 21 days. Data are presented as mean volume ± SEM.

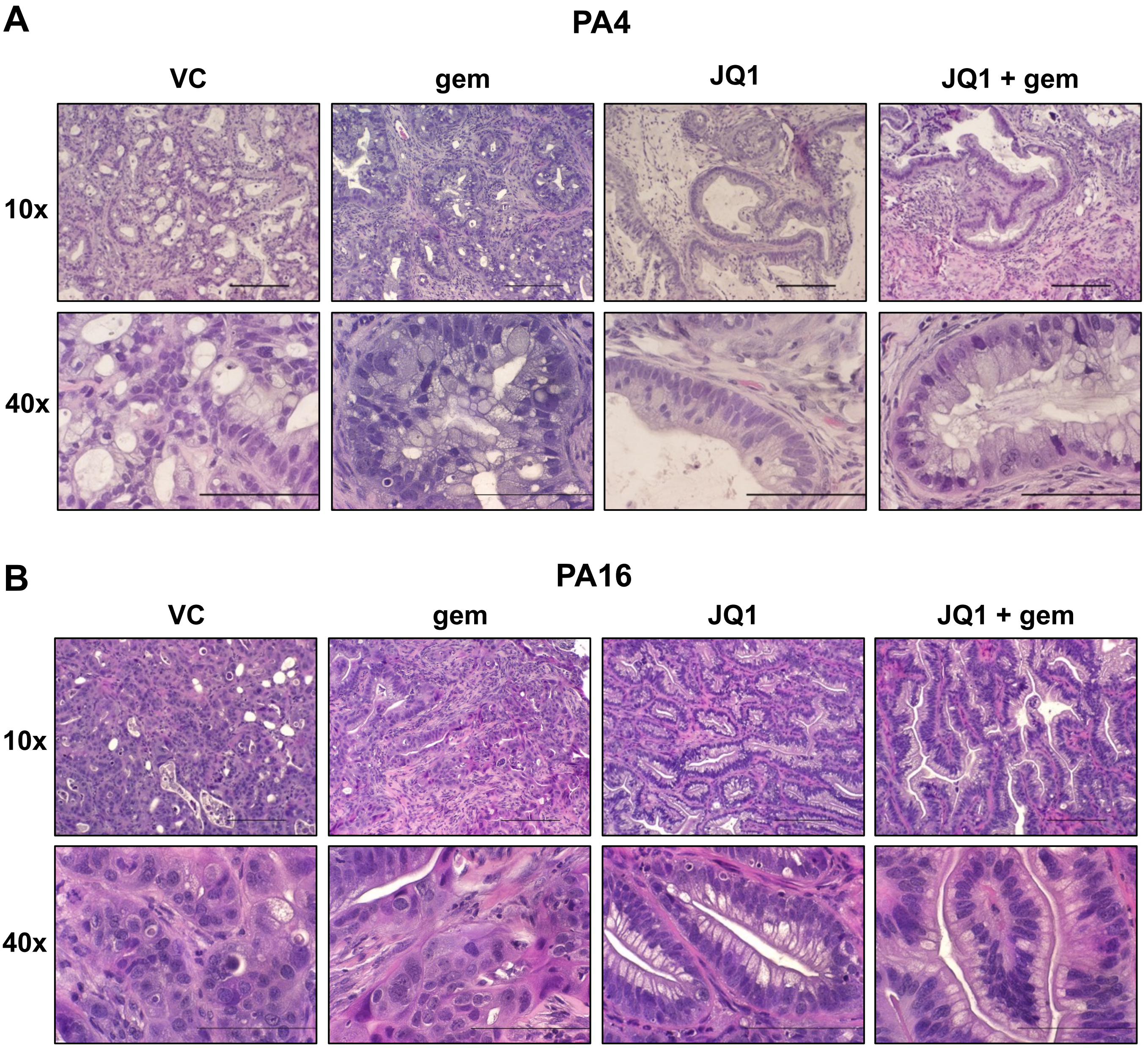

Figure S2. JQ1 and JQ1 + gemcitabine induce tumor cell differentiation *in vivo*. (**A**) PA4 and (**B**) PA16 tumors exposed to JQ1 or JQ1 + gemcitabine *in vivo* show a more well differentiated morphology than untreated (VC) tumors. Treated tumors had intact nuclear polarity, less nuclear stratification and pleomorphism, compared to untreated tumors. Scale bar 10x = 20 μm and 40x = 10 μm.

A

PA4 Ingenuity Canonical Pathways	P-value
Taurine Biosynthesis	2.5e-3
L-Cysteine Degradation	5.0e-3
Pentose Phosphate Pathway (Non-oxidative Branch)	7.5e-3
Pentose Phosphate Pathway	1.2e-2
Endocannabinoid Neuronal Synapse Pathway	1.5e-2
Extrinsic Prothrombin Activation Pathway	2.0e-2
CREB Signaling in Neurons	3.9e-2
Coagulation System	4.3e-2
Superpathway of Methionine Degradation	4.6e-2
Intrinsic Prothrombin Activation Pathway	5.1e-2

PA16 Ingenuity Canonical Pathways	P-value
Aryl Hydrocarbon Receptor Signaling	1.5e-5
Inhibition of Matrix Metalloproteases	1.5e-5
Bladder Cancer Signaling	3.5e-5
RAR Activation	1.0e-4
Glioblastoma Multiforme Signaling	1.5e-4
Tumor Microenvironment Pathway	2.1e-4
Senescence Pathway	2.9e-4
Xenobiotic Metabolism CAR Signaling Pathway	3.3e-4
Pancreatic Adenocarcinoma Signaling	3.9e-4
Atherosclerosis Signaling	4.8e-4

B

	PA4		PA16	
	**Gene ID**	**Fold change (VC/JQ+gem)**	**Gene ID**	**Fold change (VC/JQ+gem)**
1	DNAJC25-GNG10	25134.3	ABCB1	18.6399
2	LOC100101148	168.865	NOTUM	18.1146
3	FGB	98.9564	LINC00513	11.3258
4	TMIGD2	13.1942	LINC02365	9.59563
5	ZNF256	11.5449	CLDN18	9.42094
6	ART1	9.89564	ATF3	8.0513
7	FAM172BP	8.24637	LYZ	7.67766
8	CDO1	8.24637	PTGIS	7.34076
9	LOC100507389	8.24637	THRIL	6.82499
10	NPY6R	6.5971	SCARA3	6.48858

Figure S3. Pathways and gene products upregulated by JQ1 + gemcitabine *in vivo* in PA4 or PA16 models of PDAC. (A) Pathways upregulated by JQ1 + gemcitabine *in vivo* in PA4 or PA16 PDX models of PDAC. **(B)** Ten gene products upregulated *in vivo* by JQ1 + gemcitabine compared to vehicle controls (VC) in PA4 or PA16 tumors.

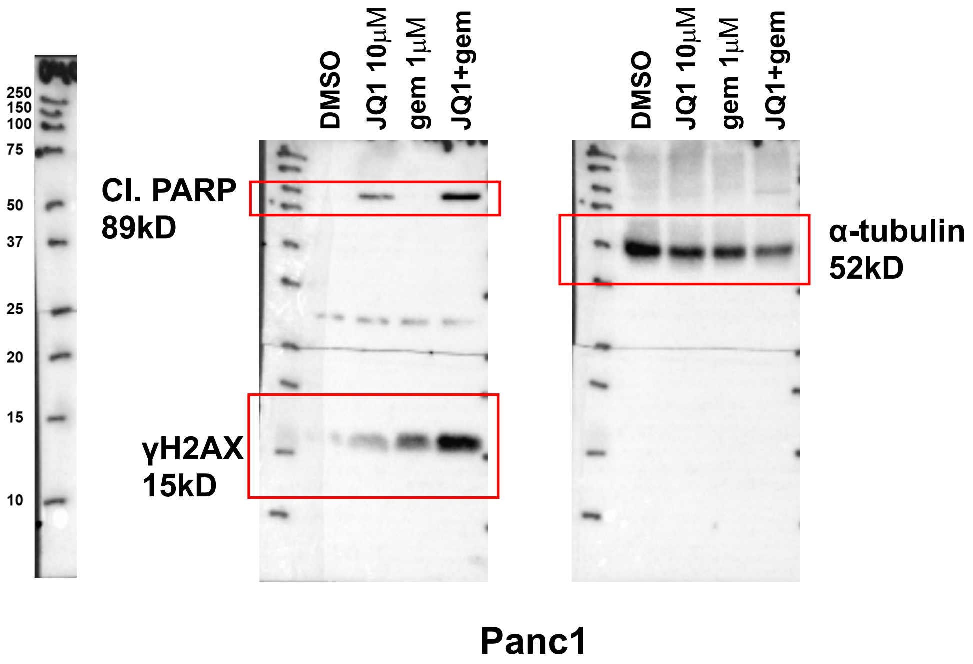

Panc1 Densitometry			
	α-tubulin	γH2AX	Cl. PARP
DMSO	33449.10	551.33	594.96
JQ1	22611.87	10528.31	7754.66
gem	25234.66	21964.03	618.38
JQ1+gem	21589.11	40968.66	20107.10

Figure S4. The whole uncropped immunoblot images with densitometry intensity/reading analysis of Figure 2D.

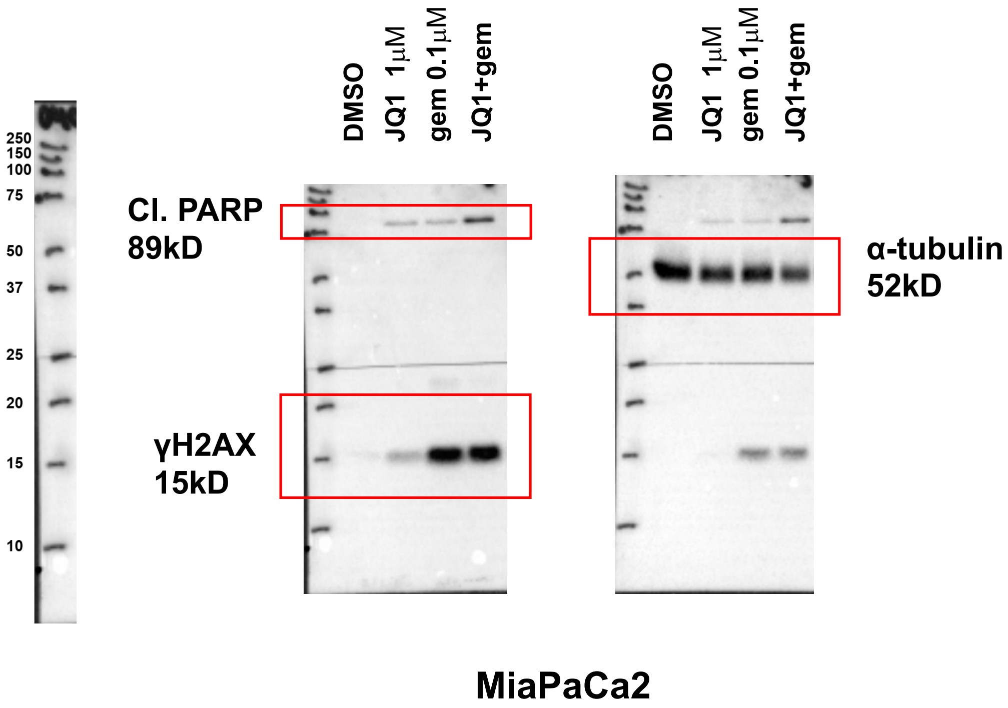

MiaPaCa2 Densitometry			
	**α-tubulin**	**γH2AX**	**Cl. PARP**
**DMSO**	59905.3	1395.52	416.36
**JQ1**	50889.33	9118.80	5497.43
**gem**	53591.35	38898.63	5588.66
**JQ1+gem**	45363.88	42292.24	19023.47

Figure S5. The whole uncropped immunoblot images with densitometry intensity/reading analysis of Figure 2E.

Primers	HMGCS2	Fwd 5′-AGAGCTTCTCCCCGTGAATC-3′<br>Rev 5′-CCATAAGCCCAGGACAGTGA-3′
	APOC1	Fwd 5′-TGGTTCTGTCGATCGTCTTG-3′<br>Rev 5′-TGTCCTCCAGTGTGTTTCCA-3′
	GAPDH	Fwd 5′-AACATCATCCCTGCTTCCAC-3′<br>Rev 5′-GACCACCTGGTCCTCAGTGT-3′

Table S1. Primer sets used to perform qRT-PCR.

		Fold change (VC/JQ + gem)		
	**Gene ID**	**PA4**	**PA16**	**Average**
1	*HMGCS2*	-5.82927	-5.91705	-5.8732
2	*APOC1*	-8.19664	-3.06895	-5.6328
3	*CGBS*	-7.18476	-3.20816	-5.1965
4	*NEGR1*	-2.30404	-7.92216	-5.1131
5	*PNCK*	-6.14989	-3.21169	-4.6808
6	*TAC3*	-5.65906	-2.91972	-4.2894
7	*ARL4C*	-4.08678	-3.89347	-3.9901
8	*GBP4*	-2.29105	-3.51424	-2.9026

Table S2. Eight gene products downregulated *in vivo* by JQ1 + gemcitabine compared to vehicle controls (VC) in PA4 and PA16 tumors.

**LXR/RXR activation pathway genes altered by JQ1 + gemcitabine *in vivo***		
	**PA4**	**PA16**
APOCI	-8.2	-3.1
APOC2	-3.9	-1.1
APOE	-1.8	-4.4
AGT	-1.3	-2.6
ALB	-5.6	1
CD14	-4.9	-2.2
ILIR2	-3.1	-3
PLTP	-4.6	-2.6
PON1	-1.8	-3.2
SERPINF1	-1.1	-2.2
TLR3	-2.5	-1.4
TLR4	-3.4	1
TTR	1	-3.4

Table S3. Gene products in the LXR/RXR activation pathway that are altered by JQ1+gemcitabine compared to vehicle controls in PA4 and PA16 tumors.
